# Polystyrene–Br End-Group Modification via Electrolysis: Adjusting Hydrogenation vs Coupling Selectivity

**DOI:** 10.1021/acsmacrolett.5c00053

**Published:** 2025-03-07

**Authors:** Alessandro Zampieri, Felix Schnaubelt, Khidong Kim, Giovanni Lissandrini, Marco Fantin, Krzysztof Matyjaszewski, Christian Durante, Abdirisak A. Isse

**Affiliations:** † Department of Chemical Sciences, University of Padova, Via Marzolo 1, 35131 Padova, Italy; ‡ Institute of Physical Chemistry and Center for Materials Research, Justus Liebig University Giessen, Heinrich-Buff-Ring 17, 35392 Giessen, Germany; § Center for Macromolecular Engineering, Carnegie Mellon University, 4400 Forbes Avenue, Pittsburgh, Pennsylvania 15213, United States

## Abstract

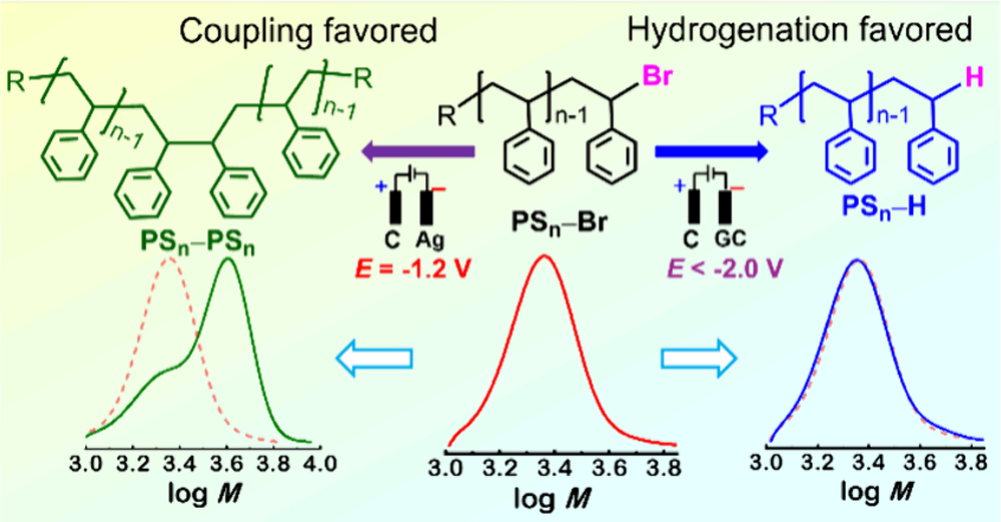

Atom transfer radical polymerization (ATRP) enables the precise synthesis of polymers with well-defined architectures, controlled molecular weights, and low dispersity. However, the halogen end-groups inherent to ATRP polymers can pose challenges due to their chemical reactivity and thermal instability. To address these issues, various strategies, including chemical and photochemical methods, have been developed for chain-end modification. This study introduces an electrochemical approach to selectively reduce halogen end-groups in ATRP polymers. Using glassy carbon (GC) and silver electrodes, the reductive cleavage of C–Br in bromine-capped polystyrene was investigated. Cyclic voltammetry revealed that polystyrene-bromide undergoes electron transfer accompanied by the concerted removal of the C–Br functionality. The Ag electrode facilitated electrocatalysis with enhanced activity. Controlled-potential electrolysis demonstrated that reaction conditions, particularly the choice of proton donors, significantly influence product distribution, enabling selective hydrogenation or dimerization of polystyrene-bromide chain ends. This work advances the understanding of electrochemical strategies for tailoring polymer end-group functionality.

Atom transfer radical polymerization (ATRP) is a powerful technique enabling the synthesis of polymers with well-defined architectures, predetermined molecular weights, low dispersity and high chain-end fidelity.^1,2^ A fundamental prerequisite of ATRP is the establishment of an equilibrium between propagating radicals, P_*n*_^•^, and dormant species, P_*n*_–X, mediated by a catalytic system via atom transfer. Therefore, the obtained polymer carries an ω-halogen end-group, which may be undesirable in many applications because of the chemical reactivity of the carbon–halogen moiety and its thermal instability.^3−7^ TGA analysis of halogen-terminated polymers showed a two-step thermal weight loss pattern in which degradation of the end-group occurs in the first step at 150–250 °C.^5−8^

Halogen chain ends in ATRP polymers can be removed via chemical transformations to achieve desirable chain-end functionalities.^9−15^ Reductive coupling with stoichiometric copper catalysts that are commonly used in ATRP has also been reported.^16,17^ The reduction of the carbon–halogen bond to a terminal C–H group has been extensively studied. Common methods include using trialkyltin hydride, an efficient H atom donor, and cumene as an alternative donor.^18,7^ Reduction of the C–X chain end by hydrogen gas over Pd catalyst supported on carbon has been reported.^19^ This approach requires mild reaction conditions and was successfully applied to a series of polymers, including polystyrene, polyacrylates, and poly(acrylic acid). A light-mediated method for the removal of terminal halogen chain ends was also reported.^20,21^ Using 10-phenylphenothiazine as a reducing photoredox catalyst, different families of both soluble and surface-grafted polymers, prepared by ATRP, were hydrodehalogenated. Last, reduction with stoichiometric amounts of N-heterocyclic carbene boranes has recently been reported.^22^

In this paper, we present an electrochemical approach for the chain-end reduction of halogen-terminated polymers. Indeed, electrochemistry is a powerful tool in selective transformations and has recently been shown to be applicable to both polymer modifications and degradations.^23,24^ The reductive cleavage of carbon–halogen bonds in organic halides has been widely investigated for synthetic applications including electrosynthesis of hydrodehalogenated products and in the remediation of halogenated pollutants.^25−27^ Overall, the electroreduction process involves the reduction of RX to a radical R^•^, which may be further reduced to the corresponding carbanion R^–^. Whether radical or carbanionic chemistry is triggered depends on several factors, including the nature of the electrode material, applied potential, and chemical structure of the organic halide. Many types of electrode materials have been used, but the most recent research in the field focused on silver as the best electrocatalytic material for the reductive cleavage of carbon–halogen bonds.^27−29^

In this study, silver and glassy carbon (GC) electrodes were used, the latter being a noncatalytic material functioning as an outer-sphere electron donor.^30^ Bromine-capped polystyrene (PS_*n*_–Br, where *n* = number of monomer units) prepared by ATRP and (1-bromoethyl)benzene, used as a simpler model substrate, were investigated. Conditions favoring either selective hydrogenation of the terminal C–Br group or reductive coupling of polystyrene chains were identified. The method benefits from mild conditions, adjustable selectivity, and high atom economy with nearly 100% faradaic efficiency.

## Cyclic Voltammetry

**Figure 1:**
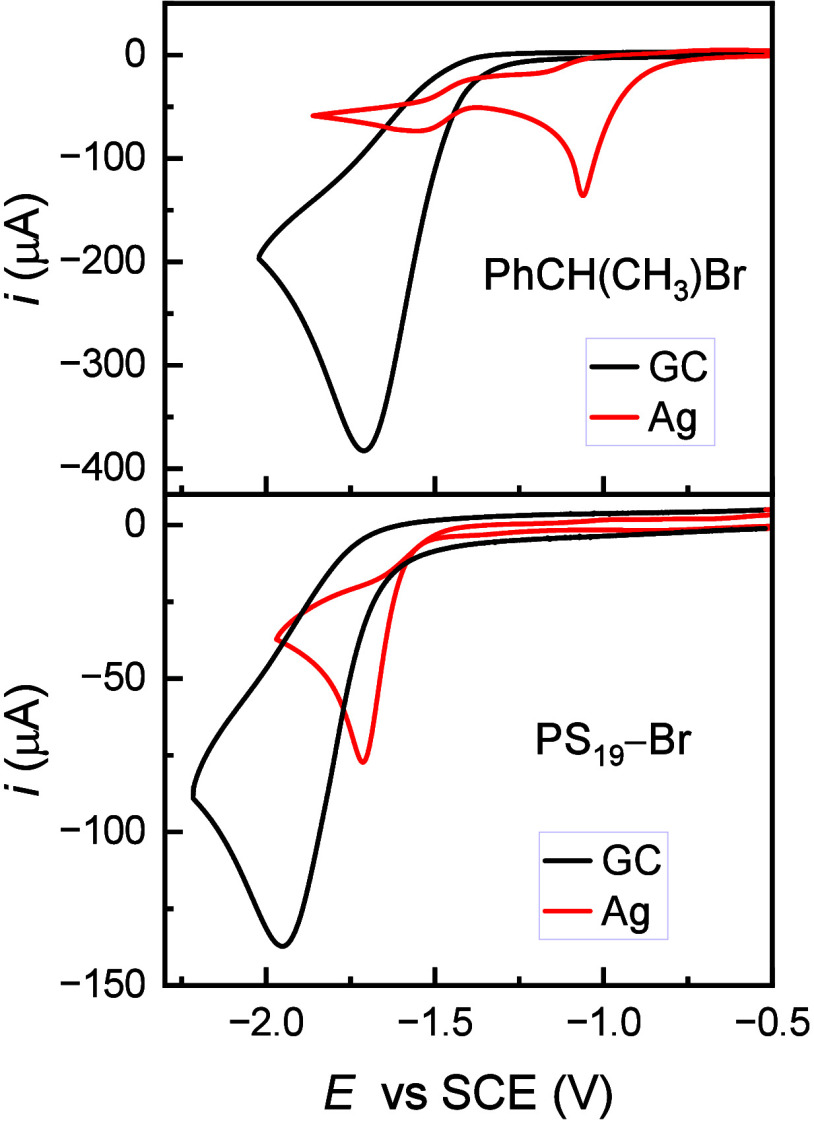
Cyclic voltammetry of 10 mM PhCH(CH_3_)Br and 10 mM PS_19_–Br (*M*_n_ = 2150 g/mol) recorded on GC (*A* = 0.07 cm^2^) and Ag (*A* = 0.03 cm^2^) electrodes in DMF + 0.1 M Et_4_NBF_4_ at *v* = 0.2 V/s and 25 °C.

Figure 1 shows cyclic voltammetry (CV) of (1-bromoethyl)benzene (PhCH(CH_3_)Br) and PS_19_—Br (*M*_n_ = 2150 g/mol, measured by GPC) on both GC and Ag electrodes. On GC, both compounds exhibit a single irreversible cathodic peak with a quite negative peak potential (*E*_p_ < −1.70 V vs SCE). Although the terminal group of brominated polystyrene is similar to PhCH(CH_3_)Br, the latter is more easily reduced than the polymer (Figure 1). This difference may be attributed to a higher electron donor ability of the polymer chain than a methyl group and/or increased distance of the C–Br moiety from the electrode surface. It is well-known that increasing the distance of closest approach of a redox substrate to the electrode, due to bulkiness of the substrate or electrolyte, results in a reduced rate of electron transfer, i.e., higher overpotential.^31−33^

The peak potentials shift cathodically as the scan rate, *v*, is increased. Plots of *E*_p_ vs log *v* show straight lines with a slope d*E*_p_/dlog*v* ≈ −100 mV (Figure S4), which gives electron transfer coefficients, α, of about 0.3 (Table 1). This value is typical of the electroreduction of alkyl halides, which undergo concerted electron transfer (ET)/bond breaking.^34^ Thus, for both PhCH(CH_3_)Br and PS_19_–Br the process at GC occurs by a concerted dissociative ET yielding R^•^ and X^–^, as previously reported (eq 1).^28,29,35^ Benzyl and benzylic-type radicals have redox potentials between −1.40 V and −1.64 V vs SCE.^36,37^ These values are more positive than *E*_p_ of RBr at GC so that R^•^ is further reduced to R^–^ as soon as it is formed at the electrode (eq 2).
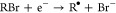
1
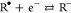
2

**Table 1:** Electrochemical Data for the Reductive Cleavage of C–Br in (1-Bromoethyl)benzene and Polystyrene–Br in DMF + 0.1 Et_4_NBF_4_ at 25 °C

Substrate	Electrode	*E*_p_^a^ (V)	∂*E*_p_/∂ log *v* (mV)	α^b^
PhCH(CH_3_)Br	GC	–1.70	–99	0.30
PhCH(CH_3_)Br	Ag	–1.06	–108	0.27
PS_19_–Br^c^	GC	–1.95	–102	0.29
PS_19_–Br^c^	Ag	–1.71	–110	0.27

a Peak potentials measured vs saturated calomel reference electrode at *v* = 0.2 V/s.

b Calculated from *∂E*_p_/∂ log *v* = −1.15*RT*/α*F*, where *R* is the universal gas constant and *F* is Faraday constant.^40^

c Prepared by ATRP, *M*_n_ = 2150 g/mol (Figure S1).

At Ag, two cathodic peaks of unequal height are observed for PhCH(CH_3_)Br, whereas PS_19_–Br shows a single irreversible reduction peak. The first reduction peak of PhCH(CH_3_)Br and the cathodic peak of the polymer are anodically shifted by 0.64 and 0.24 V when compared to GC (Table 1). This positive shift is due to the well-documented electrocatalytic activity of Ag toward the reductive cleavage of carbon–halogen bonds.^29,38^ Since electrocatalysis at Ag involves the interaction of both carbon and Br atoms with the metal surface, the observed lower activity for PS_19_–Br may be due to nonoptimal surface interaction of C–Br caused by the steric effect of the bulky polymer chain. The reduction peaks at both electrodes shift cathodically with increasing *v*, and *E*_p_ vs log *v* analysis provides α = 0.27 (Table 1, Figure S4), indicating that a concerted dissociative ET takes place also at Ag (eq 1). *E*_p_ of the polymer is more negative than the redox potential of R^•^; thus, the overall process at Ag is expected to be 2e^–^ reduction of the C–Br bond at potentials near −1.7 V. In contrast, CV of PhCH(CH_3_)Br shows a cathodic peak at −1.06 V vs SCE, where R^•^ reduction cannot take place, followed by a second peak at −1.55 V vs SCE. This voltammetric pattern is much similar to that of benzyl bromide for which a detailed mechanistic analysis involving adsorption of RBr and its reduction intermediates and products, especially Br^–^, was previously reported.^35,39^ By analogy, we may assign 1e^–^ reduction of PhCH(CH_3_)Br at the first peak, followed by fast dimerization of PhCH(CH_3_)^•^; the second peak is assigned to 1e^–^ reduction of a small fraction of PhCH(CH_3_)^•^ surviving radical coupling.

## Controlled-Potential Electrolysis of PhCH(CH_3_)Br

Bulk electrolysis of PhCH(CH_3_)Br and PS_19_–Br in a divided cell was carried out at GC and Ag electrodes in the absence and presence of acetic acid (AcOH) as a proton donor. The electrolyses were stopped when the current dropped to 1% of its initial value, corresponding to almost full conversion of the starting bromide (Figure S5). The applied potential, *E*_app_, was chosen on the basis of the CV response of the substrate.

For PhCH(CH_3_)Br, the electrolysis at GC was performed near *E*_p_ of the observed single cathodic peak, whereas the potentials of both the first and second peak were used at Ag.

The principal reduction products determined by HPLC analysis were ethylbenzene and 2,3-diphenylbutane, together with styrene from side reactions (Table 2). The dimer was a mixture of meso and racemic diastereomers and was determined using the meso stereoisomer as a standard. When no acid was added, the principal product at GC was ethylbenzene (53%), accompanied by 17% dimer and 28% styrene. The product selectivity increased when the reaction was run in the presence of AcOH with an increase of ethylbenzene yield to 80% and a drastic drop of the yields of the dimer and styrene (Table 2, entries 1 and 2).

**Table 2:** Controlled-Potential Electrolysis of PhCH(CH_3_)Br and Bromine-Capped Polystyrenes in DMF + 0.1 Et_4_NBF_4_^a^

Entry	Electrode	Substrate	HA	*C*_HA_ (mM)	*E*_app_^b^(V)	*T* (h)	*n*^c^(e^–^/molecule)	RH^d^ (%)	R–R^d^ (%)	R^=^^d^ (%)
1	GC	PhCH(CH_3_)Br	-	-	–1.72	1	1.1	53	17	28
2	GC	PhCH(CH_3_)Br	AcOH	20	–1.72	1	1.7	80	5	1
3	Ag	PhCH(CH_3_)Br	-	-	–1.02	1	1.1	19	57	3
4	Ag	PhCH(CH_3_)Br	AcOH	20	–1.09	1	1.1	20	58	3
5	Ag	PhCH(CH_3_)Br	-	-	–1.49	1	1.2	25	46	5
6	Ag	PhCH(CH_3_)Br	AcOH	20	–1.58	1	1.7	51	32	2
7	GC	PS_19_–Br	-	-	–2.11	3	1.3	69	6	25
8	GC	PS_19_–Br	H_2_O	50	–2.11	5	1.2	70	4	25
9	GC	PS_19_–Br	AcOH	20	–2.10	4.3	2.1	67	3	30
10	GC	PS_19_–Br	HCO_2_H	20	–2.04	6	2.7	94	3	3
11	Ag	PS_19_–Br	-	-	–1.93	1	1.2	61	2	38
12	Ag	PS_19_–Br	H_2_O	50	–1.84	1.3	1.3	75	2	23
13	Ag	PS_19_–Br	AcOH	20	–1.66	2.5	2.4	89	2	9
14	Ag	PS_19_–Br	-	-	–1.20	20	0.9	18	63	19
15	Ag	PS_19_–Br	AcOH	20	–1.25	4.5	1.3	4	90	5
16	Ag	PS_47_–Br	AcOH	10	–1.25	22	1.9	35	63	2
17	Ag	PS_72_–Br	AcOH	10	–1.25	19	2.2	27	73	0
18	GC	PS_47_–Br	HCO_2_H	20	–2.33	5.5	3.2	95	2	2
19	GC	PS_72_–Br	HCO_2_H	20	–2.35	6	3.4	94	3	2

a General conditions: [R–Br] = 10 mM, *V* = 15 mL with PhCH(CH_3_)Br and 5 mL with PS_*n*_–Br, divided cell with a graphite anode, *T* = room temperature.

b vs SCE.

c Number of electrons consumed per molecule of R–Br, calculated as *n* = *Q*/(*FVC*_RBr_), where *Q* is the total charge, *V* is the solution volume, and *F* is Faraday constant (96485 C/mol).

d RH = PhCH_2_CH_3_ or PS_*n*_–H, R–R = coupling product, R^=^ stands for styrene or PS_*n*_^=^. Yields from HPLC for PhCH(CH_3_)Br and from ^1^H NMR and GPC with respect to initial polymer for PS_*n*_-Br, considering the chain end fidelity of the starting sample (80.6% for PS_19_–Br, 88% f or PS_47_–Br and 90% for PS_72_–Br); see example calculation in the Supporting Information.

Electrolysis at the first peak at Ag yielded 57% dimer, 19% ethylbenzene, and 3% styrene; these yields were not affected by addition of acid (Table 2, entries 3 and 4). At potentials in the region of the second peak at Ag, the electrolysis was quite sensitive to the proton availability of the medium. The principal product was 2,3-diphenylbutane with only 25% ethylbenzene when no acid was added. Conversely, when the electrolysis was performed in the presence of AcOH, ethylbenzene with a yield of 51% became the principal product, while the dimer yield dropped from 46 to 32% (Table 2, entries 5 and 6).

**Figure 2:**
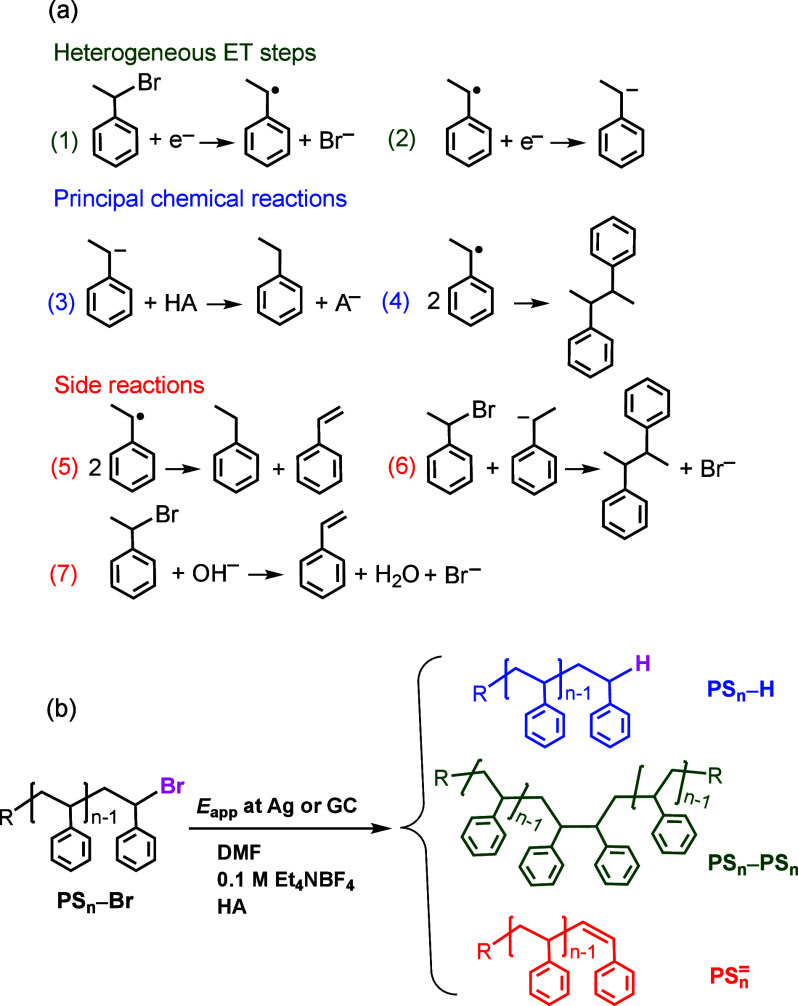
Mechanism of electrochemical reduction of (a) (1-bromoethyl)benzene and (b) Br-terminated polystyrenes at Ag and GC electrodes in DMF + 0.1 M Et_4_NBF_4_.

The results of CV investigations and bulk electrolysis of (1-bromoethyl)benzene can be rationalized by the reaction mechanism shown in Figure 2a. At *E*_app_ ≈ −1.5 V at Ag or −1.7 V at GC, both RBr and R^•^ are reduced at the electrode (eqs 1 and 2). The ensuing carbanion R^–^ is protonated by the strongest proton donor, HA, in solution, that is, AcOH or residual water when no acid is added (eq 3, Figure 2a). Formation of 2,3-diphenylbutane at GC arises from nucleophilic attack of R^–^ on PhCH(CH_3_)Br (eq 6, Figure 2a) rather than radical–radical coupling (eq 4, Figure 2a). Indeed, when a good proton donor was added, the yield of the dimer dropped from 17% to 5%.

When *E*_app_ > −1.2 V is applied at Ag, R^•^ is not readily reduced at the electrode. Thus, it can undergo typical radical reactions (eqs 4 and 5, Figure 2a). Therefore, at both electrodes the main process at very negative potentials should be conversion of RBr to RH in a 2e^–^ process, whereas mainly 2,3-diphenylbutane should be formed at Ag when less negative potentials are applied. Although these expectations were generally fulfilled, there were some complications due to side reactions.

A remarkable amount of styrene was formed at GC when no acid was added. In this experiment, PhCH_2_CH_3_ was the principal product (53%), with the residual water in DMF acting as the proton donor. Styrene was formed by the reaction of PhCH(CH_3_)Br with OH^–^ generated by protonation of R^–^ by H_2_O. This is supported by the drastic drop of styrene yield when AcOH was used as the proton donor. Additionally, independent control experiments confirmed elimination of HBr from PhCH(CH_3_)Br by the action of a base (see Supporting Information, S6). The reaction was significantly more efficient with a strong base such as (C_4_H_9_)_4_NOH compared to acetate, consistent with the observed lower yield of styrene in experiments with added AcOH (Table 2).

The process at Ag is complicated by the adsorption of RBr, R^•^, and Br^–^.^35^ Nevertheless, as expected, a high yield of dimer was obtained by electrolysis at the potentials of the first cathodic peak. A remarkable amount of dimer was formed also at *E*_app_ values around the potential of the second peak (Table 2, entries 5 and 6), where R^•^ is reducible at Ag. It appears that the Ag surface plays an important role in favoring the radical coupling reaction over hydrodehalogenation, as previously reported.^39,41−44^

It may be noted that the overall yield of PhCH(CH_3_)Br reduction products is always lower than 100%. However, the total charge passed during electrolysis matches the yields of the reduction products if one considers 2e^–^/molecule for PhCH_2_CH_3_ and 1e^–^/molecule for 2,3-diphenylbutane. The only exception is electrolysis at −1.58 V on Ag (Table 2, entry 6), where some acid reduction contributes to the observed charge. Besides styrene, small amounts of some other product(s) arising from nonelectrochemical side reactions may be formed.

## Controlled-Potential Electrolysis of PS_19_–Br

A series of electrolyses in conditions similar to those explored with the model alkyl bromide were performed on PS_19_–Br. This polymer was prepared by normal ATRP, and GPC analyses of the purified material showed *M*_n_ = 2150 g/mol and dispersity *Đ* = 1.08. ^1^H NMR analysis revealed that the starting polymer consisted of C–Br terminated chains (PS_19_–Br, 80.6%), hydrogenated chains (PS–H, 14.5%), double-bond-terminated chains (PS_19_^=^, 4.4%), and dimer chains (PS_19_–PS_19_, 0.5%) (see Supporting Information).^45,46^ Thus, only 80.6% of the starting material was reactive at the electrode surface, and yields were calculated accordingly (see Supporting Information).

**Figure 3:**
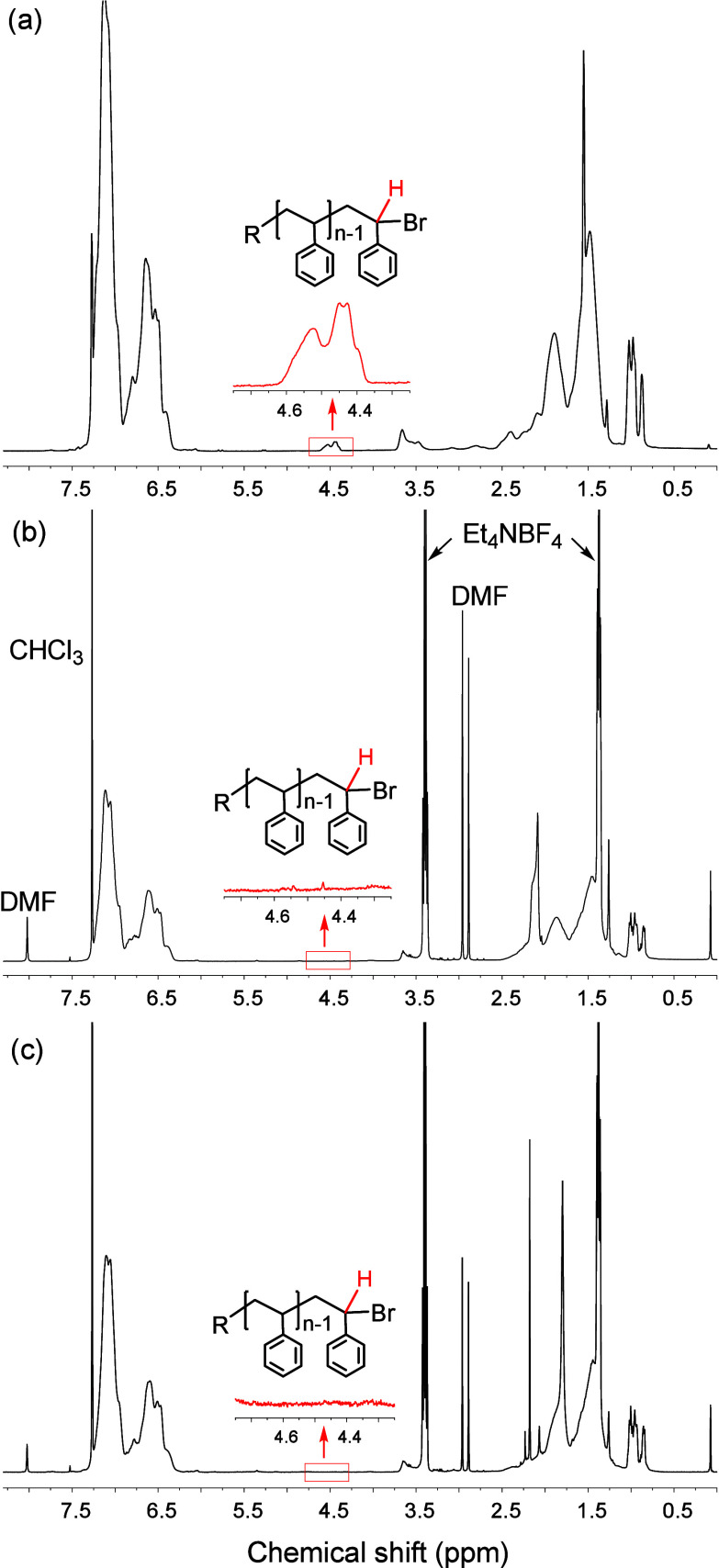
^1^H NMR spectra of PS_19_–Br in CDCl_3_ before electrolysis (a) and after electrolysis in DMF + 0.1 M Et_4_NBF_4_ carried out at −2.04 V on GC (b) or at −1.25 V on Ag (c).

Electroreduction of PS_19_–Br produced PS_19_–H, PS_19_–PS_19_, and PS_19_^=^, with product distribution strongly influenced by reaction conditions. Electrolysis was stopped after the complete reduction of terminal C–Br groups, indicated by a current drop to ∼1% of the initial value. Full conversion of PS_19_–Br was confirmed via ^1^H NMR and CV. Postelectrolysis ^1^H NMR spectra showed the disappearance of the 4.5 ppm signal, corresponding to the proton α to the terminal bromide (Figure 3), consistent with CV results (Figure S6).

**Figure 4:**
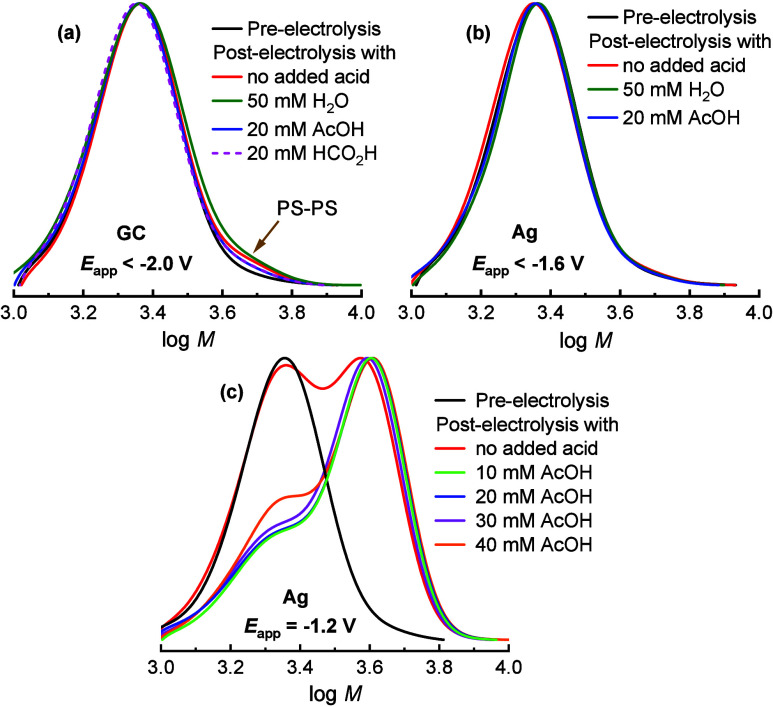
GPC traces of PS_19_–Br before and after electrolysis at GC and Ag electrodes in DMF + 0.1 M Et_4_NBF_4_ in the absence and presence of proton donors.

Electrolyses at GC were conducted at potentials 0.1–0.2 V more negative than the *E*_p_ of PS_19_–Br, and the results are summarized in Table 2. At these *E*_app_ values, the terminal C–Br bond undergoes 2e^–^ reduction to PS_19_^–^, followed by protonation to PS_19_–H. GPC traces after electrolysis were nearly identical with the initial polymer chromatogram, confirming that *M*_n_ and dispersity remained unaffected (Figure 4a). Radical–radical coupling with *M*_n_ doubling was negligible, as PS_19_^•^ was too short-lived to leave the electrode surface and dimerize.

When no effective proton donor was added, a small amount of PS_19_–PS_19_ formed due to nucleophilic substitution. Formation of PS_19_^=^, however, was more significant and arose from side reactions between PS_19_–Br and the conjugate base of the proton donor. PS_19_–H yield was ca. 70% with weak proton donors (e.g., residual or added water or acetic acid) and 94% with stronger donors like formic acid (Table 2, entries 7–10). Conversely, the yields of side products (PS_19_–PS_19_ and PS_19_^=^) decreased with the strong proton donor. Matrix-assisted laser desorption/ionization-time-of-flight (MALDI-TOF) mass spectrometry confirmed that PS_19_–H was the main reduction product at GC (Figure S14).

Charge consumption data (Table 2, column 8) showed that electrons consumed per polymer molecule (*n*) fell below the theoretical 2e^–^ value when water was used as the proton donor, due to significant nonelectrochemical side reactions. Using acetic acid or formic acid increased *n* to 2.1e^–^/molecule and 2.7e^–^/molecule, respectively, with formic acid further reducing side reactions. In both cases, minor contributions from direct acid reduction at the electrode were observed (Figure S7).

Controlled-potential electrolysis of PS_19_–Br at Ag, targeting either hydrodehalogenation or radical–radical coupling by changing *E*_app_ (Figure S8), yielded the results summarized in Table 2, entries 11–15. At *E*_app_ ≤ −1.66 V, where both PS_19_–Br and PS_19_^•^ are readily reduced, high yields of hydrogenated polymer and moderate to low yields of PS_19_^=^ were observed, with minimal formation of coupling product (Figure 4b). As with the GC electrode, the product distribution depended on the proton donor’s acidity, with stronger acids favoring PS_19_–H formation over PS_19_^=^. Formic acid was unsuitable due to its reduction at Ag within the same potential window as PS_19_–Br reduction (Figure S9). However, acetic acid provided satisfactory results, yielding a polymer with 89% C–H terminal groups while retaining the original *M*_n_ and *Đ* values after C–Br removal.

At both GC and Ag, the electrolysis time at *E*_app_ ≤ −1.66 V depended on reaction conditions such as presence of acid and type of electrode and its wetted surface area but was always ≤6 h (Table 2, column 7).

In CV at Ag, PS_19_–Br lacks a defined reduction peak before −1.7 V (Figure 1). Nonetheless, electrolysis proceeded at −1.2 V, where benzyl radicals are not reducible. The reaction proceeded with a low current and was left running overnight to reach full conversion (Figure 3c). It is likely that at this potential the reaction proceeds on a limited number of catalytic sites active for PS–Br reduction. The main product was the dimer, alongside small amounts of PS_19_^=^ and PS_19_–H. The dimer was observed via both GPC (Figure 4c) and MALDI-TOF (Figure S15). Even at these potentials (0.2–0.4 V more positive than *E*^0^ of benzyl radicals), a fraction of adsorbed PS_19_^•^ was reduced to PS_19_^–^, followed by protonation to give PS_19_–H. Surprisingly, adding acetic acid (AcOH) accelerated the reaction and improved dimer yield to 90% while limiting PS_19_^=^ and PS_19_–H formation (Table 2, entries 14 and 15). Product distribution remained stable with [AcOH]/[PS_19_–Br] ratios of 0.5–2 but showed reduced dimer yield at higher ratios (Table S1, Figure S10). GPC traces for −1.2 V experiments (Figure 4c) revealed bimodal distributions, with low MW polymers PS_19_–H and PS_19_^=^ (it should be noted that the starting polymer already contained some unreactive PS_19_^=^ and PS_19_–H).

## Electrolysis of Higher *M*_n_ Polystyrenes

Two other bromine-terminated polystyrenes, PS_47_–Br (*M*_n_ = 5100 g/mol) and PS_72_–Br (*M*_n_ = 7700 g/mol), were also studied (Table 2, entries 16–19). These polymers, with higher purity and chain-end fidelity than PS_19_–Br (88–90% chain-end fidelity), exhibited similar voltammetric behavior, showing a single irreversible cathodic peak comparable to PS_19_–Br (Figure S11). Lower peak currents were attributed to reduced diffusion coefficients for higher molecular weights.

At Ag, a broad peak near −1.3 V suggested 1e^–^ reduction of PS_*n*_–Br to PS_*n*_^•^, followed by radical coupling. Electrocatalysis on Ag at −1.25 V fully removed the terminal C–Br group, forming PS_*n*_–PS_*n*_ (63–73%), though small amounts of PS_*n*_–H and PS_19_^=^ persisted (Figure S12). As previously observed for PS_19_–Br, the reactions were quite slow.

At GC, electrolysis at potentials more negative than the peak potential achieved excellent hydrodebromination via 2e^–^ reduction also for these higher MW samples, as shown by monomodal GPC traces identical with those of the initial brominated polymer (Figure S13). The process exhibited high selectivity, yielding 94–95% PS_*n*_–H (Table 2, entries 18 and 19). The reaction time at this electrode was about 6 h, as in the case of PS_19_–Br, and did not show dependence on polymer *M*_w_.

In conclusion, the electrochemical reductive removal of PS_*n*_–Br was studied at GC and Ag electrodes, with products characterized by NMR and GPC, in comparison with the model compound (1-bromoethyl)benzene. Complete removal of the C–Br bond could be readily achieved on both inert GC and electrocatalytic Ag electrodes. The product distribution was controlled by the choice of electrode material, applied potential, and proton availability of the medium. At the inert GC electrode with *E*_app_ < −1.7 V vs SCE, hydrodebrominated PS_*n*_–H was obtained in high yields (94–95%) within a few hours in the presence of formic acid. At the Ag electrode, applied potentials more positive than the reduction peak yielded moderate to high amounts of the coupling product, PS_*n*_–PS_*n*_ (63–90%), with reaction times under 6 h in the presence of acetic acid. Lower molecular weight polymer samples produced higher dimer yields.

## Supporting Information Available

Materials and methods, experimental procedures, and additional experimental and characterization data (PDF)
