# Supplementary material for: Polystyrene–Br End-Group Modification via Electrolysis: Adjusting Hydrogenation vs Coupling Selectivity
Source: ACS Macro Lett. 2025 Mar 7;14(3):364–70. doi: 10.1021/acsmacrolett.5c00053 (PMC11924314; doi:10.1021/acsmacrolett.5c00053)
Supplement: Supplementary file 1 — mz5c00053_si_001.pdf [file mz5c00053_si_001.pdf]

Supporting Information for:

Polystyrene-Br End-Group Modification via Electrolysis: Adjusting Hydrogenation vs Coupling Selectivity

Alessandro Zampieri,[a] Felix Schnaubelt,[a,b] Khidong Kim,[c] Giovanni Lissandrini,[a] Marco Fantin,[a]* Krzysztof Matyjaszewski,[c] Christian Durante,[a] Abdirisak A. Isse[a]*

[a]Department of Chemical Sciences, University of Padova, Via Marzolo 1, 35131, Padova, Italy

[b]Institute of Physical Chemistry and Center for Materials Research, Justus Liebig University Giessen, Heinrich-Buff-Ring 17, 35392, Giessen, Germany

[c]Center for Macromolecular Engineering, Carnegie Mellon University, 4400 Forbes Ave, Pittsburgh, PA, USA

S1. Materials

N,*N*-Dimethylformamide (DMF, Carlo Erba Reagents, HPLC grade, 99.9%), tetrahydrofuran (THF, Carlo Erba Reagents, HPLC grade, 99.9%), deuterated chloroform ($CDCl_3$, Sigma Aldrich, 99.8% atom D), dichloromethane (Carlo Erba Reagents, RS - for HPLC - Isocratic Grade), methanol (Sigma Aldrich, ≥ 99.8%), ethanol (Carlo Erba Reagents), acetic acid (Sigma Aldrich, ≥ 99.8%), formic acid (Carlo Erba Reagents, 99%), acetonitrile (Carlo Erba Reagents, RS - for HPLC - Isocratic Grade), tris[2-(dimethylamino)ethyl]amine (Me_6TREN, Ambeed, 97%), bis(2-dimethylaminoethyl)methylamine (PMDETA), copper(I) bromide (CuBr, Sigma-Aldrich. 99.999%), copper(II) bromide ($CuBr_2$, Aldrich, 99.999%), 2-hydroxyethyl α-bromoisobutyrate (TCI Chemicals, > 97.0%), ethyl α-bromoisobutyrate (EBiB, Sigma-Aldrich, 98%), tin ethyl hexanoate ($Sn(EH)_2$, Sigma-Aldrich), (1-bromoethyl)benzene (Sigma-Aldrich, 97%) ethylbenzene (Sigma-Aldrich, for synthesis) and meso-2,3-diphenylbutane (Aldrich) were used as received. Styrene (Sigma-Aldrich, 99.9%) was purified by passing through a basic alumina column. Tetraethyl tetrafluoroborate (Et_4NBF_4, Aldrich, ≥ 98%) was recrystallized from ethanol and dried in a vacuum oven at 50 °C for 48 h.

S2. Instrumentation

Electrochemical experiments were carried out at room temperature in a 3-electrode cell under a blanket of argon gas. The working electrode, which was always cleaned/activated at the beginning of each experiment, was either Ag or glassy carbon (GC), while the reference electrode was an AgI-coated silver wire immersed in 0.1 M Bu_4NI in DMF ($Ag/AgI/I^-$) and the counter electrode was a graphite rod in a separate compartment (no electrolysis were attempted in an undivided cell). The working electrodes (a 3-mm GC disk or 1-mm Ag disk) used in cyclic voltammetry were cleaned by polishing with a 0.25-μm diamond paste, followed by ultrasonic rinsing in ethanol for 5 min. In electrolysis experiments, a GC plate or a silver wire was used. The GC plate was cleaned in the same way as the GC disk, whereas the Ag wire was activated by dipping in a solution of nitric acid,

followed by rinsing in MilliQ water and acetone. The reference electrode was calibrated at the end of each experiment by adding ferrocene to the solution and measuring its standard potential by cyclic voltammetry. This allowed converting potentials measured versus $Ag/AgI/I^-$ to the saturated calomel electrode (SCE) scale by using $E^{\mathrm{o}}_{\mathrm{Fc^+/Fc}}$ = 0.476 V vs SCE.[1]

Voltammetric experiments were performed with an Autolab PGSTAT302N potentiostat/galvanostat, interfaced to a PC running NOVA software (Metrohm AG, The Netherlands), whereas an EG&G Princeton Applied Research 173 potentiostat/galvanostat equipped with a 179 digital coulometer was used for controlled-potential electrolysis.

Matrix-assisted laser desorption/ionization-time of flight ToF-MALDI was carried out with an AB SCIEX 4800 MALDI TOF/TOF. Dithranol was used as a MALDI matrix. Sodium trifluoroacetate (NaTFA) was used as a cationization agent. The dithranol (60 mg/ mL), polystyrene sample (15 mg/mL), and cationization agent (10 mg/mL) were dissolved in THF. These solutions were mixed at a volume ratio of dithranol:polymer:cationization agent = 20:10:3. 1 mL of the mixture solution was deposited on a MALDI sample plate, and the spots were dried in air at room temperature.

The products of (1-bromoethyl)benzene electrolysis were analyzed using a JASCO 2075 HPLC, equipped with a UV detector set at 260 nm. A 25 cm × 4.6 mm 4 µm Hydro-RP 80 Å, C18 LC Column (Phenomenex, Synergi™) was used. The eluent (1 mL/min) was a mixture of acetonitrile and MilliQ water, acidified with 5 g/L acetic acid. Authentic compounds were used to identify and quantify the products.

The starting bromine-capped polystyrene (PS_n-Br) and its electrolysis products were analyzed by gel permeation chromatography (GPC). An Agilent 1260 instrument equipped with a refractive index detector (RID) and two PSS Gram columns (dimension: 300 × 8 mm; particle size: 10 mm) was used. The detector and columns were thermostated at 50 °C and 60 °C, respectively, and the eluent was DMF + 10 mM LiBr at a flow rate of 1 mL/min. The analysis was based on a calibration curve performed with 12 linear poly(methyl methacrylate) standards of molecular weight M_n = 540 – 2,210,000 (Agilent EasiVial). Both PS_n-Br and its reduction products, dissolved in $CDCl_3$, were also analyzed by ^{1}H NMR with a Bruker Avance III 400 MHz spectrometer.

S3. Procedures

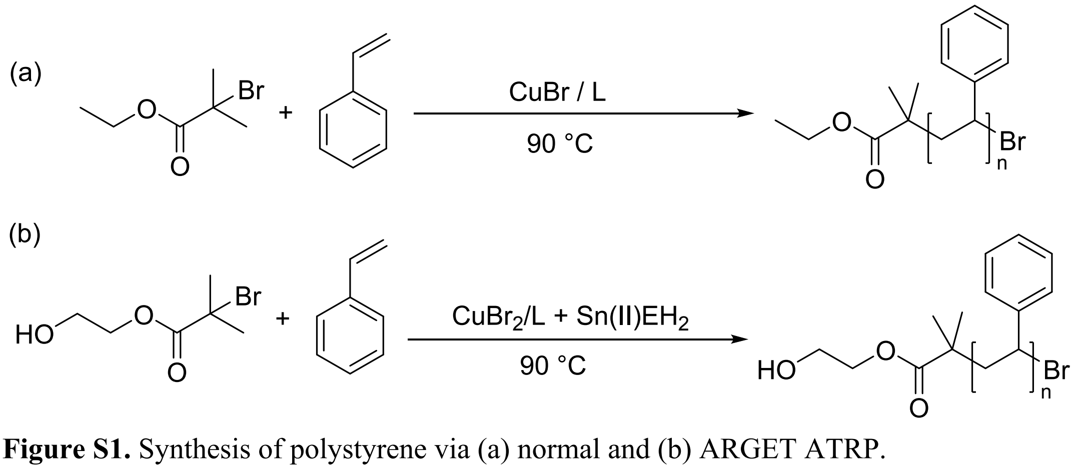

Figure S1. Synthesis of polystyrene via (a) normal and (b) ARGET ATRP.

S3.1. PS_{19}-Br Synthesis via Normal ATRP

- Styrene preparation: 150 mL styrene filtered over basic alumina to remove stabilizer.
- Schlenk flask setup:
  - 15.0 mL (0.131 mmol) styrene added to a dried 50 mL Schlenk flask with a stir bar.
  - Degassed with three vacuum/argon cycles and an additional 5-minute vacuum degassing.
  - Saturated with argon.
- Reagent addition:
  - 400 µL (1.94 mmol) PMDETA and 2.72 mmol EBiB added to the styrene.
  - Solution degassed as before.
- Copper catalyst preparation:
  - 314 mg (2.19 mmol) CuBr and 21 mg (0.0095 mmol) $CuBr_2$ added to a second dried Schlenk flask and degassed with three vacuum/argon cycles.
- Polymerization:
  - Styrene solution transferred via cannula to the CuBr/$CuBr_2$ flask (green dispersion).
  - Degassed again, stirred, and heated in an 80 °C oil bath for 3 h 15 min.

Purification

- Reaction quenching: Cooled to room temperature and quenched with oxygen.
- Filtration:
  - Diluted with 20 mL dichloromethane (DCM) and filtered through neutral alumina to remove copper salts.
  - Alumina column washed twice with 20 mL DCM.
- Solvent removal: Rotary evaporated at 50 °C under vacuum to leave a slightly yellow residue.
- Precipitation:
  - Residue mixed with 50 mL MeOH to form a white suspension.
  - Stored at -15 °C overnight to precipitate the product.

S3.2. PS_{47}-Br e PS_{72}-Br synthesis via ARGET ATRP

Polystyrene was synthesized using bulk ATRP via ARGET ATRP (Figure S1). Initial styrene concentration was 8.2 M, with 45 ppm of copper and a reaction temperature of 90 °C. The molar ratio was [HO-EBiB]:[Styrene]:[Me_6Tren]:[CuBr]:[$Sn(EH)_2$] = 1:100:0.08:0.005:0.08.

Procedure:

- **Preparation:** 100 mL of styrene was filtered through basic alumina to remove stabilizers. Three stock solutions were prepared: (1) CuBr and Me_6Tren in styrene, (2) HO-EBiB in styrene, and (3) $Sn(EH)_2$ in styrene. A Schlenk flask was evacuated and filled with N_2 three times over 20 minutes. Styrene and stock solutions were deoxygenated separately under N_2.
- **Reaction:** Degassed styrene (4 mL), initiator (0.35 mmol), catalyst (1.74 µmol), ligand (27.8 µmol), and $Sn(EH)_2$ (27.8 µmol) were sequentially added to the flask. The reaction mixture, containing 45.3 ppm of CuBr, was heated at 90°C in an oil bath.

Purification Procedure

1. The reaction flask was cooled to room temperature, and the reaction quenched with oxygen.

2. The mixture was diluted with 20 mL DCM and filtered through neutral alumina, followed by washing the column twice with 20 mL DCM.
3. The filtrate was concentrated via rotary evaporation (50°C, 200 mbar) for ~1 hour.
4. Methanol (50 mL) was added to the residue, forming a white, cloudy suspension. After refrigeration overnight, the precipitate settled, and the supernatant was decanted.
5. The precipitate was dissolved in 5 mL DCM, re-precipitated with methanol (50 mL), and dried under vacuum (50 °C) for two days to yield a white powder/crystal-like product.

3.3. Typical procedure for controlled-potential electrolysis of (1-bromoethyl)benzene

The electrochemical cell was assembled with the electrodes and an inert gas line, and charged with Et_4NBF_4 (0.325 g, 1.5 mmol) and 15 mL of DMF under Ar flow. (1-Bromoethyl)benzene (0.0278 g, 0.15 mmol) was then added and a CV was recorded. If the experiment was to be performed in the presence of a proton donor, the required amount of acid was added and again a CV was recorded. The working electrode was switched from 1 mm or 3 mm disk electrode to a big area electrode and electrolysis was started by applying a constant potential near the first or second cathodic peak at Ag or in correspondence of the only cathodic peak observed at GC. The electrolysis was stopped when the initial current dropped to 1% of its initial value, indicating exhaustive conversion of the starting organic halide, which was confirmed by CV recorded at the end of electrolysis as well as by HPLC analysis.

3.4. Typical procedure for controlled-potential electrolysis of PS_n-Br

The electrochemical cell with electrodes, solvent and electrolyte was prepared as previously described. The only difference was a smaller solution volume, 5 mL rather than 15 mL. The polymer (0.05 mmol) was added and after degassing a CV was recorded. In experiments with a proton donor, the required amount of acid was added and again a CV was recorded. A small sample (0.1 mL) was withdrawn for GPC analysis and the remaining solution was electrolyzed at a fixed potential, chosen depending on the type of working electrode. Usually, the electrolysis was exhaustive and was stopped when the initial current dropped to 1%. At the end of electrolysis, a sample (0.1 mL) was taken for GPC analysis. Samples for GPC analysis were diluted 10 times in THF and filtered through neutral alumina and a polytetrafluoroethylene (PTFE) membrane of 0.2 mm pore size.

3.5. Characterization of PS_n-Br and its electrolysis products

The amount of Br-terminated chains was calculated by ^{1}H-NMR, comparing the signal of the H atom in proximity to the C-Br bond (E) to the CH_2 signals of the initiators (D) (Fig. S2).

$$\text{Amount of C-Br functionality} = \frac{2A_E}{A_D} = \frac{2}{2.48} \times 100 = 80.6\%$$

The amount of coupling product (PS_n-PS_n) was evaluated by analyzing the bimodal GPC chromatogram of the electrolyzed solution (Fig. S3). Deconvolution of the GPC curve provides separate curves for PS_n-H and PS_n-PS_n and a comparison between the areas of the peaks of PS_n-PS_n ($A_{\mathrm{PS_n-PS_n}}$) and initial PS_n-Br ($A_{\mathrm{PS_n-Br}}$) allows estimation of the yield of PS_n-PS_n ($Y_{\mathrm{PS_n-PS_n}}$) as:

$$Y_{PS_n-PS_n}\ (\%) = \frac{A_{PS_n-PS_n}}{A_{PS_n-PS_n} + A_{PS_n-Br}} \times 100$$

The starting polymer PS_{19}-Br already contained a small amount of coupled chains (0.5%), which was subtracted from the yield of PS_n-PS_n displayed in the main text.

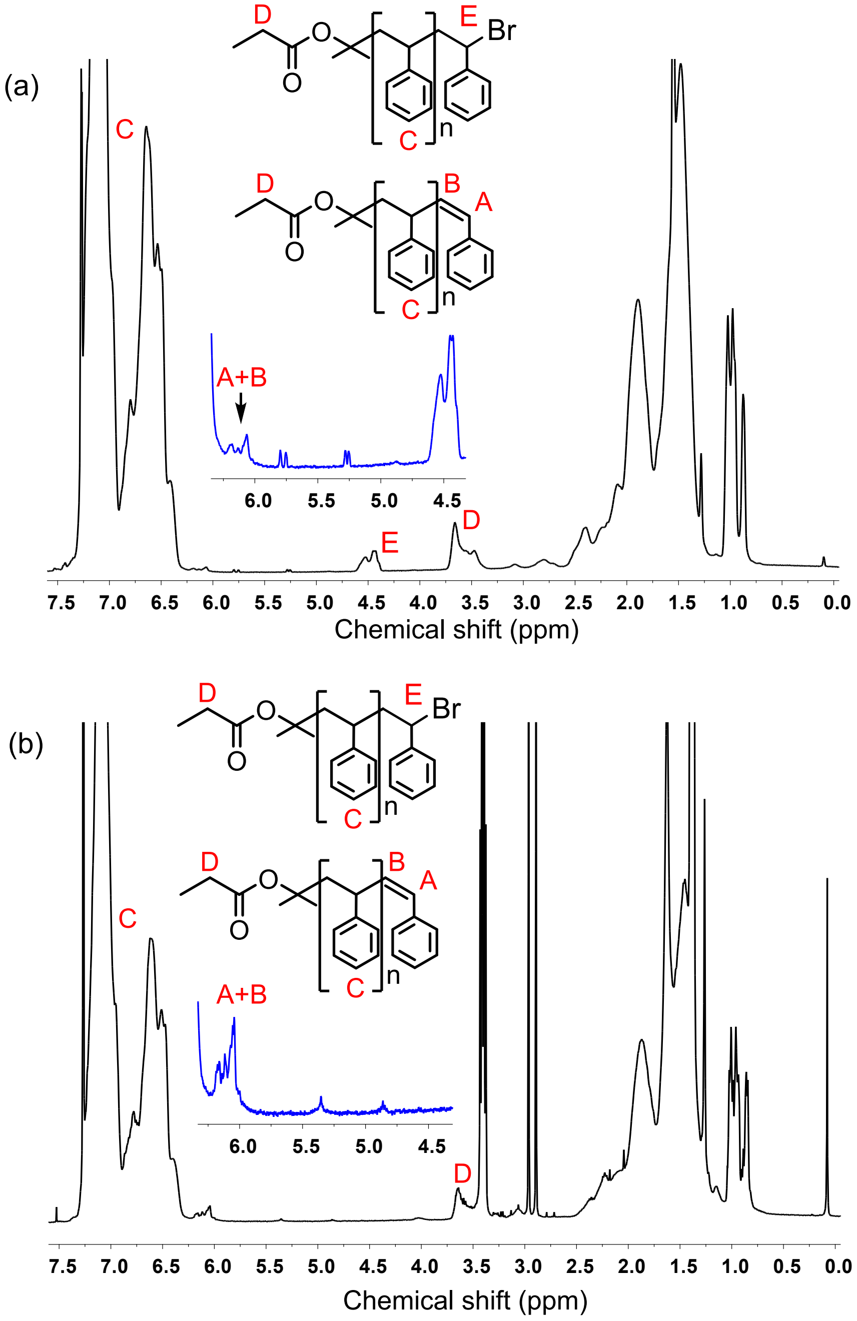

Figure S2. ^{1}H NMR spectra of PS_{19}-Br (a) before and (b) after electrolysis at Ag at -2.09 V vs SCE in DMF + 0.1 M Et_4NBF_4 in the presence of 5-fold excess of H_2O.

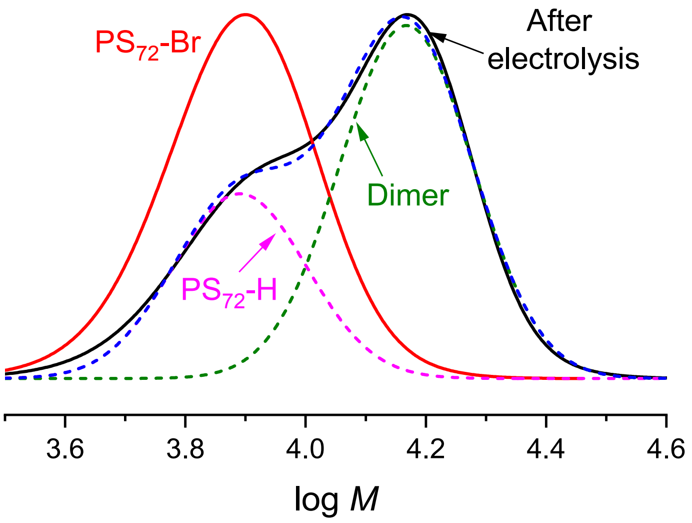

Figure S3. GPC of 10 mM PS_{72}-Br before (red line) and after (black line) electrolysis at -1.25 V vs SCE at Ag in DMF + 0.1 M Et_4NBF_4 in the presence of one equivalent of acetic acid. The dashed lines are the separate and combined traces after deconvolution.

The yield of polymer chains ending with a double bond (PS_n(-H)) was estimated by ^{1}H NMR. As shown in Fig. S2a, the starting polymer contained a small fraction of PS_n(-H), which can be determined by the integrals of peaks D of the initiator and A+B of the polymer containing the terminal double, PS_n(-H). For example, in the case of PS_{19}(-H):

$$\mathrm{PS_n}(-\mathrm{H})\ \% = \frac{A_{\mathrm{A+B}}}{A_{\mathrm{D}}} \times 100 = 4.4\%$$

This amount of PS_n(-H) present in the starting material was subtracted from the observed final amount of product to obtain the final yield of PS_n(-H).

In the spectrum of the electrolyzed solution (Fig. S2, the signal for the two methylene protons of the initiator (D) partially overlaps a signal due to protons of the electrolyte. This precludes the use of signal D as a reference system. However, the multiplet C of the aromatic protons of the polymer, which does not change during electrolysis, can be used as an internal standard. For PS_{19}-Br, the ratio between the integrals of signals C and A+B in the starting polymer is $A_{\mathrm{C}}{:}A_{\mathrm{A+B}} = 125{:}0.11$. Thus, if the integration of the spectrum is performed with a value of 125 for C, the area of A+B will be 0.11. This allows defining a relationship between the area of signal A+B and the percentage of PS_n(-H), $Y_{\mathrm{PS_n}(-\mathrm{H})}$, present in the polymer. Since the number of aromatic protons will not change during electrolysis, the spectrum of the product mixture can be integrated using 125 for the area of the multiplet C. Then the following proportionality holds:

$$\frac{0.11}{125}{:}4.4 = \frac{A_{\mathrm{A+B}}}{125}{:}Y_{\mathrm{PS_n}(-\mathrm{H})}$$

and the yield PS_n(-H) is given by

$$Y_{\mathrm{PS_n}(-\mathrm{H})} = \frac{A_{\mathrm{A+B}}}{0.11} \times 4.4 = 40A_{\mathrm{A+B}}$$

After the determination of the fractions of PS_n-PS_n and PS_n(-H) present in the electrolysis mixture, the remaining fraction was assumed to be made of hydrodebrominated polymer, PS_n-H.

S4. Cyclic voltammetry of (1-bromoethyl)benzene and PS_{19}-Br

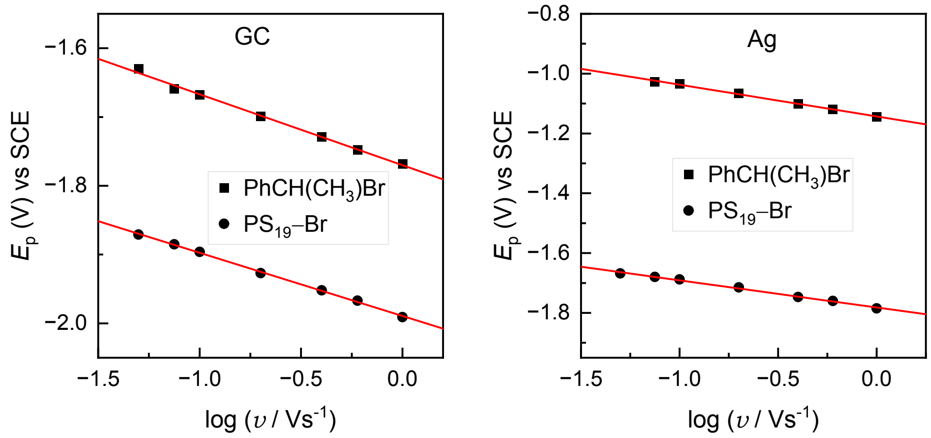

Figure S4. Linear variations of cathodic peak potentials of (1-bromoethyl)benzene and PS_{19}-Br in DMF + 0.1 M Et_4NBF_4.

S5. Cyclic voltammetry of (1-bromoethyl)benzene before and after controlled-potential electrolysis

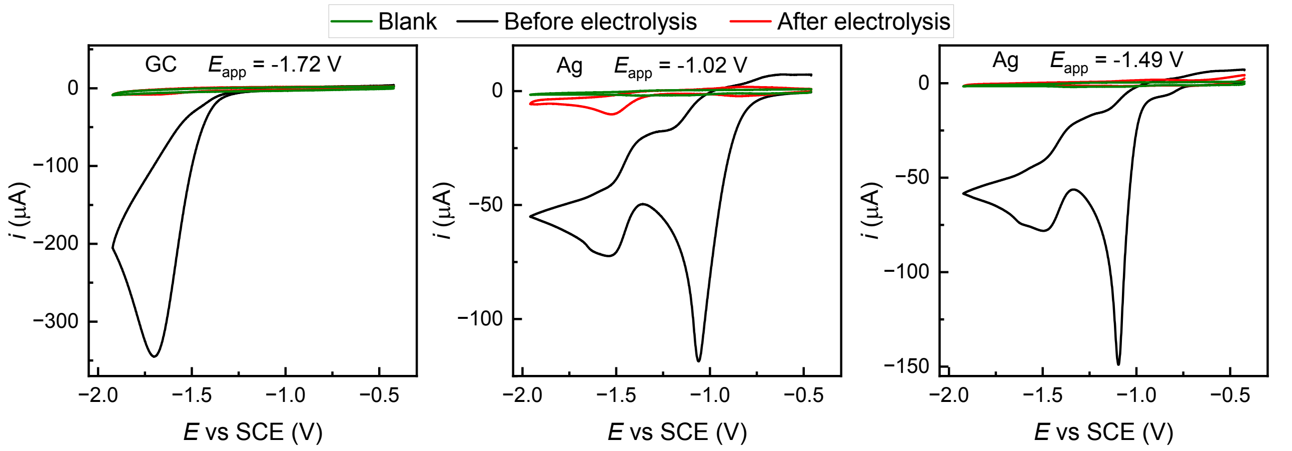

Figure S5. Cyclic voltammetry of 10 mM (1-bromoethyl)benzene in DMF + 0.1 M Et_4NBF_4, recorded at GC and Ag electrodes at $\upsilon = 0.2$ V s^{-1} before and after controlled-potential electrolysis.

S6. Hydrodebromination of (1-bromoethyl)benzene

A mixture of (1-bromoethyl)benzene and tetrabutylammonium hydroxide 0.01 M each in DMF + 0.1 M Et_4NBF_4 was prepared in a flask and left stirring for 30 minutes. The reaction mixture was then analyzed by HPLC, which showed that 97% of (1-bromoethyl)benzene has reacted yielding 52% styrene. The missing quantity of (1-bromoethyl)benzene was assumed to be converted to $PhCH(CH_3)OH$, which could not be observed in HPLC, by nucleophilic substitution of Br^- with OH^-.

A similar experiment was run using as a base tetrabutylammonium acetate prepared by mixing equimolar amounts of acetic acid and tetrabutylammonium hydroxide. Again high conversion of (1-bromoethyl)benzene (91%) was observed after 30 minutes of reaction. In this case however the yield of styrene was quite low (9%).

These experiments confirm that styrene can be formed by the action of a base on (1-bromoethyl)benzene. Conditions favorable for such a reaction are met during electrolysis as electrogenerated R^- is protonated by residual water or by AcOH in experiments with added acid. Predictably, the yield of styrene was highest in electrolysis at GC without added acid. Indeed, in that experiment more than 50% of (1-bromoethyl)benzene was reduced to ethylbenzene via $2e^-$ reduction of the bromide, followed by protonation of R^- by H_2O. This sequence of reactions produced a high concentration of OH^-, which readily reacted with the starting alkyl bromide.

S7. Controlled-potential electrolysis of PS_{19}-Br

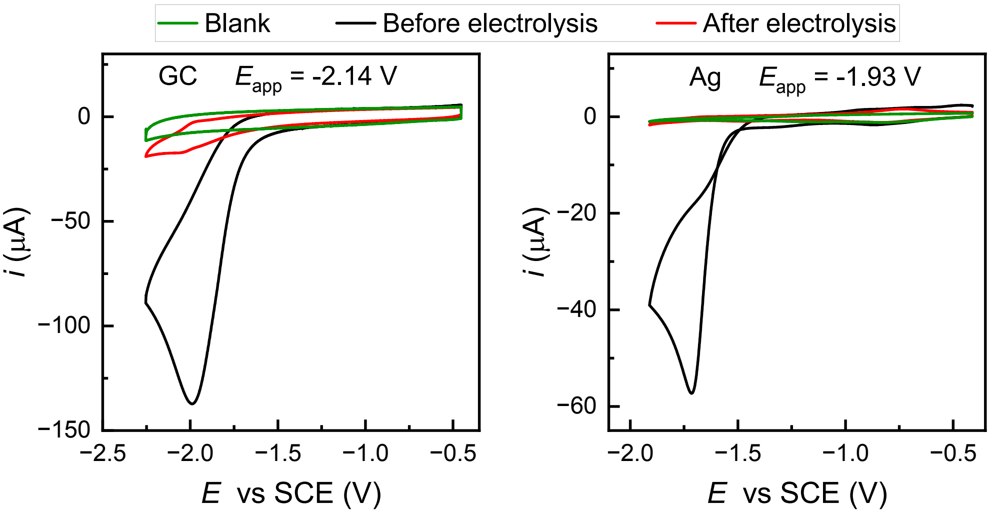

Figure S6. Cyclic voltammetry of 10 mM PS_{19}-Br at GC and Ag electrodes in DMF + 0.1 M Et_4NBF_4, recorded at υ = 0.2 V/s before and after controlled-potential electrolysis.

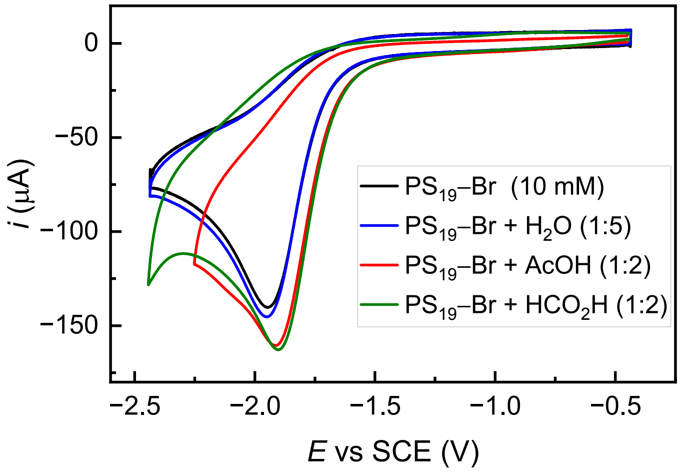

Figure S7. Cyclic voltammetry of PS_{19}-Br at GC in DMF + 0.1 M Et_4NBF_4, recorded at υ = 0.2 V/s in the absence and presence of added acids.

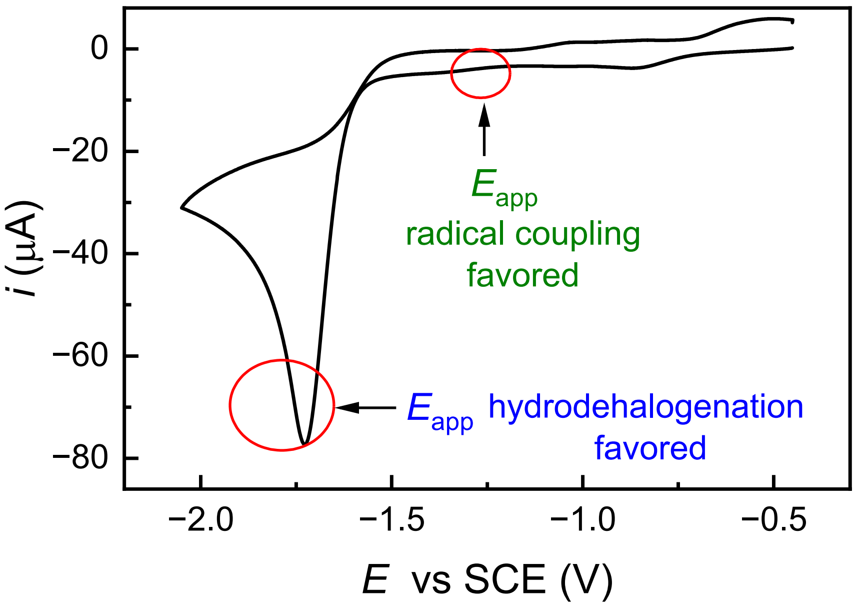

Figure S8. Cyclic voltammetry of 10 mM PS_{19}–Br in DMF + 0.1 M Et_4NBF_4, recorded at Ag electrodes at υ = 0.2 V/s. The red circles indicate the potential windows used for controlled-potential electrolysis.

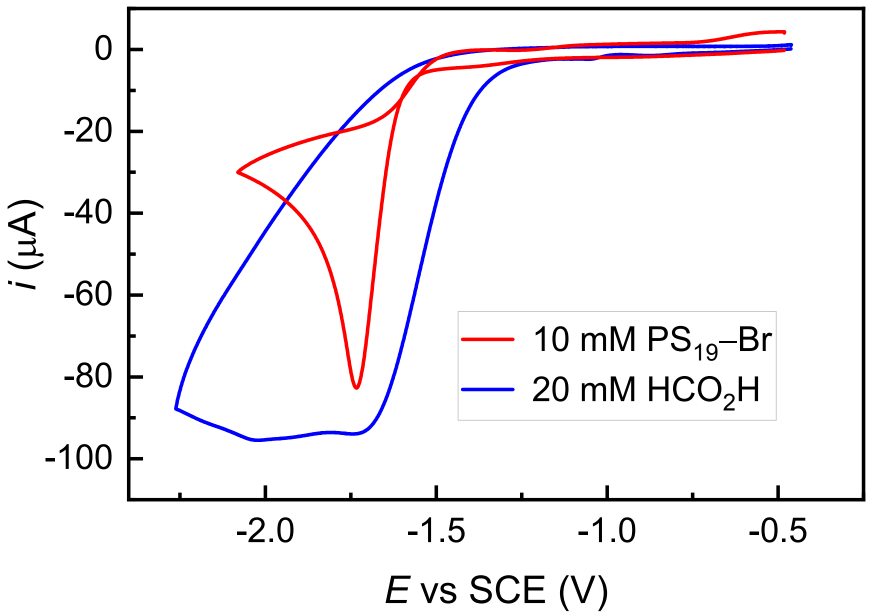

Figure S9. Cyclic voltammetry of PS_{19}–Br and formic acid in DMF + 0.1 M Et_4NBF_4, recorded at Ag at υ = 0.2 V/s.

S8. Effect of acetic acid concentration on distribution of PS_{19}–Br reduction products

Table S1. Controlled-potential electrolysis of 10 mM PS_{19}–Br in DMF + 0.1 Et_4NBF_4 in the presence of acetic acid at different concentrations.[a]

Entry	electrode	HA	C_{HA} (mM)	E_{app}[b] (V)	n[c] (F/mol)	PS[d] (%)	PS-PS[d] (%)	PS(-H)[d] (%)
1	Ag	AcOH	5	-1.26	1.1	23	72	5
2	Ag	AcOH	10	-1.20	1.1	19	73	8
3	Ag	AcOH	20	-1.25	1.3	18	72	9
4	Ag	AcOH	30	-1.10	1.1	19	66	7
5	Ag	AcOH	40	-1.10	1.2	21	63	10

[a]General conditions: [PS_{19}–Br] = 10 mM, V = 5 mL, divided cell with a graphite anode, T = room temperature. [b]vs SCE. [c]Number of electrons consumed per molecule of PS_{19}–Br, calculated as $n = Q/(F\bullet V\bullet C_{RBr})$, where Q is the total charge, V is the solution volume and F is Faraday constant (96485 C/mol). [d]Yields from ^{1}H NMR were calculated with respect to converted PS_{19}–Br.

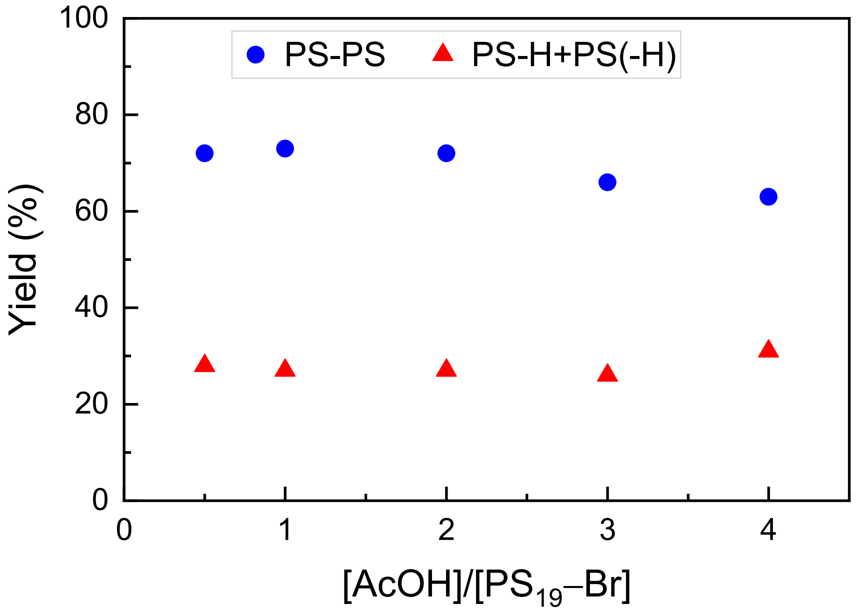

Figure S10. Controlled-potential electrolysis of 10 mM PS_{19}–Br on Ag at $E_{app} \approx$ -1.2 V vs SCE in the presence of different amounts of acetic acid.

S9. Bromine removal from high M_n polystyrenes

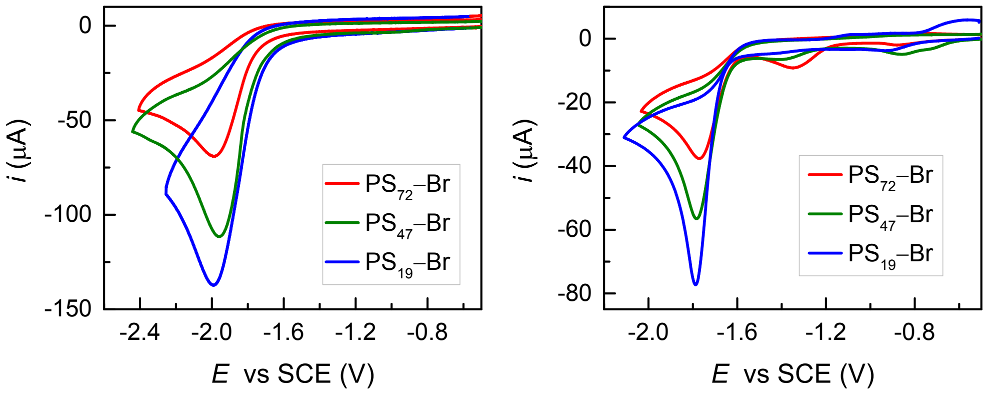

Figure S11. Cyclic voltammetry of 10 mM PS_n–Br of different molecular weights (PS_{19}–Br, M_n = 2150 g/mol; PS_{47}–Br, M_n = 5100 g/mol; PS_{72}–Br, M_n = 7700 g/mol), recorded on GC and Ag electrodes in DMF + 0.1 M Et_4NBF_4 at υ = 0.2 V/s.

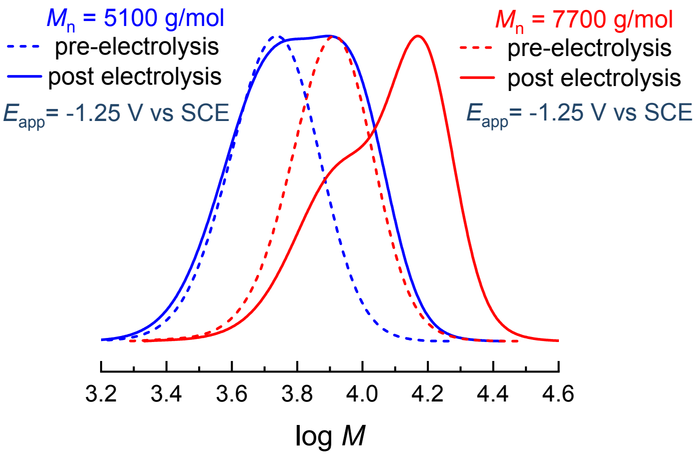

Figure S12. GPC traces of PS_{47}–Br and PS_{72}–Br before and after electrolysis at Ag in DMF + 0.1 M Et_4NBF_4 in the presence of acetic acid; [PS_n–Br] = 10 mM, [AcOH] = 10 mM.

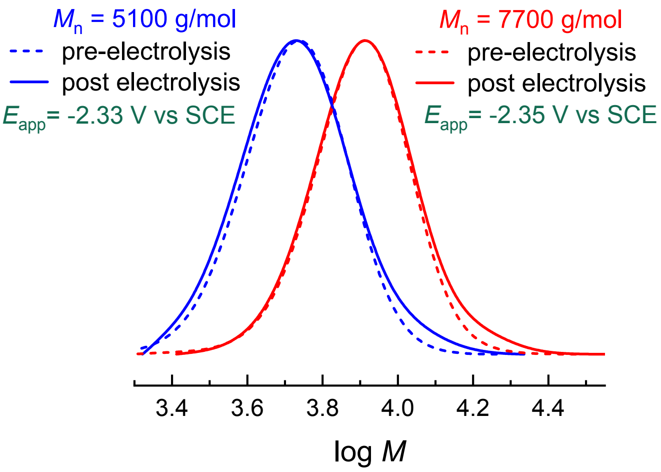

Figure S13. GPC traces of PS_{47}–Br and PS_{72}–Br before and after electrolysis at GC in DMF + 0.1 M Et_4NBF_4 in the presence of formic acid as a proton donor. [PS_n–Br] = 10 mM, [HCO_2H] = 20 mM.

S10. Maldi TOF

Bromine-capped polystyrenes undergo elimination during ionization so that PS_n(-H)Na^+ was by far the main ion observable in the MALDI TOF spectra (Fig. S14), in agreement with literature reports that support dehydrobrobination upon ionization.[2]

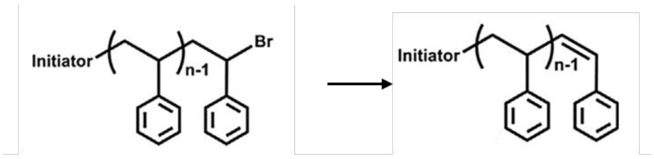

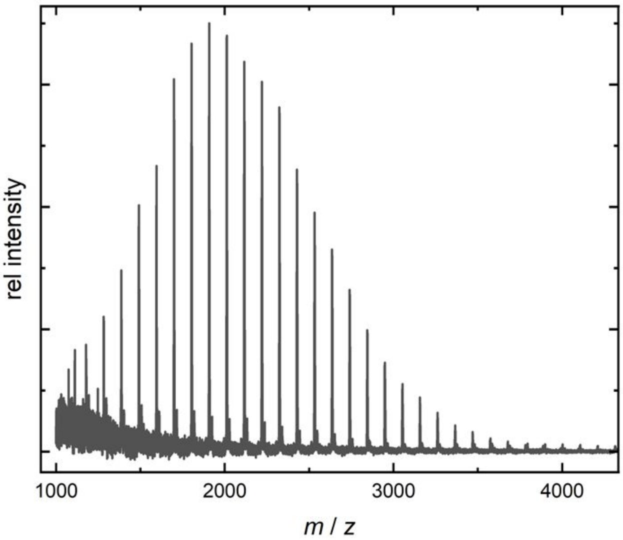

Figure S 14. MALDI spectrum of PS_{19}-Br.

Under conditions favorable for hydrodebromination (GC at -2.1 V vs SCE with acid, Entry 3, Table 3), only the reduced product PS_n-H(Na^+) was observed in the MALDI spectrum, with a moleculareight higher than that of PS_n(-H) by two atomic mass units (Fig. S15). No peaks associated with PS_n(-H) or PS_n-OH were observed in the MALDI spectrum.

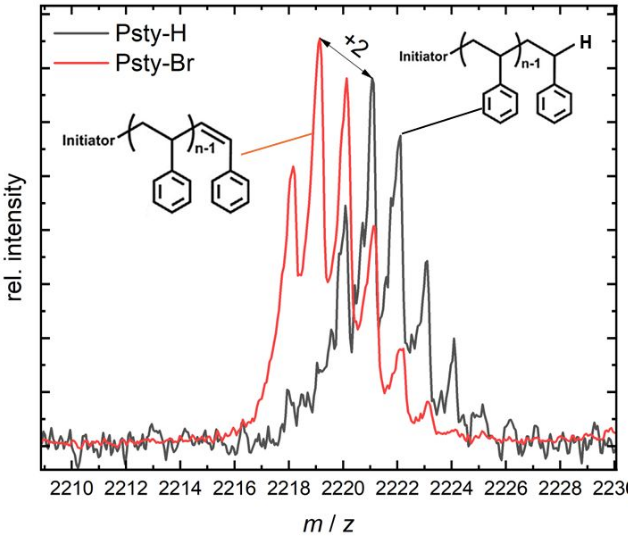

Figure S14. Zoomed comparison of the MALDI measurement of PS_{19}–Br and the electrolysis product on GC at -2.1 V vs SCE in the presence of acetic acid, revealing that only PS_{19}-H was observed.

Under conditions favorable for dimerization, after electrolysis on Ag electrode at -1.2 V vs SCE in the presence of AcOH (Entry 9, Table 3), a clear evidence of doubling molecular weight was observed (Fig. S16).

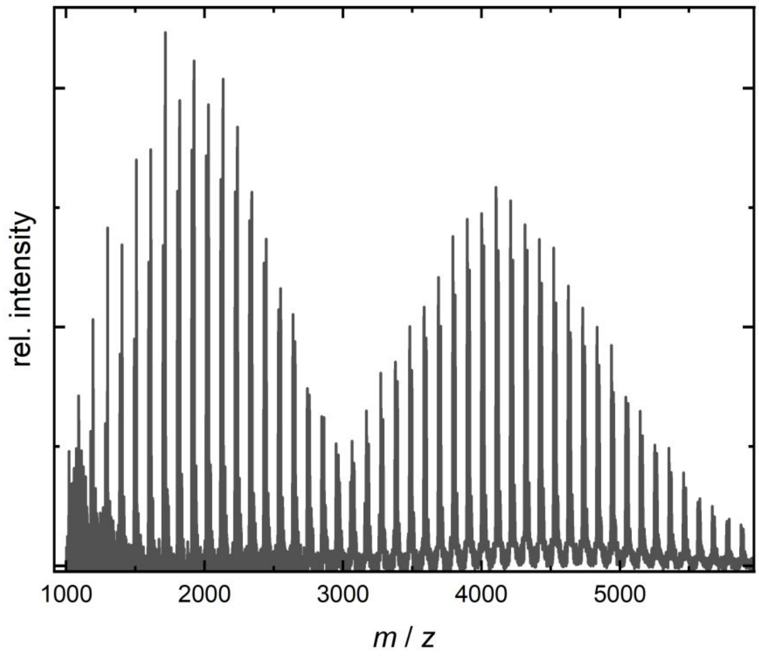

Figure S15. MALDI spectrum of the coupled product obtained from electrolysis of 10 mM PS_{19}–Br on Ag at -1.20 V vs SCE with 40 mM acetic acid.

References

1) Falciola, L.; Gennaro, A.; Isse, A. A.; Mussini, P. R.; Rossi, M. *J. Electroanal. Chem.* **2006**, 593, 47-56.
2) Kim, K.; Hasneen, A.; Paik, H.; Chang, T. *Polymer* **2013**, 54, 6133e6139
